# Promiscuous Binding in a Selective Protein: The Bacterial Na^+^/H^+^ Antiporter

**DOI:** 10.1371/journal.pone.0025182

**Published:** 2011-10-12

**Authors:** Raphael Alhadeff, Assaf Ganoth, Miriam Krugliak, Isaiah T. Arkin

**Affiliations:** Department of Biological Chemistry, The Alexander Silberman Institute of Life Sciences, The Hebrew University of Jerusalem, Jerusalem, Israel; Russian Academy of Sciences, Institute for Biological Instrumentation, Russian Federation

## Abstract

The ability to discriminate between highly similar substrates is one of the remarkable properties of enzymes. For example, transporters and channels that selectively distinguish between various solutes enable living organisms to maintain and control their internal environment in the face of a constantly changing surrounding. Herein, we examine in detail the selectivity properties of one of the most important salt transporters: the bacterial Na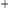/H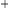 antiporter. Selectivity can be achieved at either the substrate binding step or in subsequent antiporting. Surprisingly, using both computational and experimental analyses synergistically, we show that binding *per se* is not a sufficient determinant of selectively. All alkali ions from Li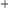 to Cs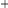 were able to competitively bind the antiporter's binding site, whether the protein was capable of pumping them or not. Hence, we propose that NhaA's binding site is relatively promiscuous and that the selectivity is determined at a later stage of the transport cycle.

## Introduction

Channels and transporters share a crucial property that underpins their physiological and biochemical functions: an ability to transport substrates in a selective manner. This ability enables cells to both protect the integrity of their content on the one hand, while communicating with the environment on the other. For example, without the ability to discriminate between Na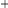 and K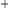 by voltage-gated channels, neural activity in our body cannot take place. Taken together, transport selectivity through the membrane is one of the fundamental prerequisites for life.

Based on the above, it is of no surprise that considerable research has gone into unraveling the precise mechanism of selectivity, and in particular - of ion channels [1]–[12]. Herein, we have focussed on the other class of proteins that are capable of facilitating selective membrane permeation: transporters, and the *Escherichia coli* Na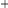/H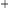 as its representative.

Na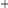/H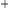 antiporting, first discovered in 1974 by Mitchell & West [13], plays a primary role in maintaining homeostasis of pH and Na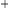 concentration, the latter having a pivotal impact on cell volume, as well. Proteins capable of performing this antiporting function can be found ubiquitously in plants, animals and microorganisms, and are present in cell cytoplasmic membranes and in the membranes of many eukaryotic organelles [14]. The first antiporter to be discovered [15] and the only one for which a structure is available [16], is the *Escherichia coli* Na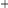/H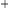 antiporter A, named NhaA. In *Escherichia coli*, NhaA is the only member of the Nha family absolutely required for survival in alkaline conditions in the presence of high external Na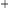 concentration [17].

Following the crystallographic structure of NhaA, solved to 3.45 Å, one can note twelve transmembrane segments (TMS I through XII) [16]. Of those TMSs, numbers IV and XI, show an uncommon structure of oppositely-oriented, discontinuous helices, *i.e.* each TMS consists of a short 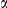-helix followed by a short unfolded segment ending with another short helix. Also seen in the x-ray structure are two funnels, leading from the bulk on both sides of the membrane to the putative binding site, D164. The cytoplasmic funnel is wide and negatively-charged, corresponding to the cation uptake path, while the periplasmic funnel is slightly narrower. The previous observations regarding the so-called TMS IV/XI assembly highlight its potential importance in the protein's function and dynamics. Lastly, the protein harbors a 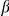-hairpin, situated in the loop connecting TMS I and II, which forms with the other loops a smooth periplasmic face, roughly parallel to the membrane's leaflet. In contrast, the cytoplasmic face features helices protruding from the parallel plane.

Previous mutagenesis experiments showed that D133, D163 and D164 are essential to NhaA's activity [18]. This finding suggested that these aspartic acid residues, adjacent to the TMSs IV/XI assembly, take part in the transport of ions along the antiporter. Therefore, the movement of Na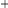 ions out of the vestibules was examined under different protonation states of D163 and D164. Recent molecular dynamics (MD) simulations on *Escherichia coli's* NhaA have suggested a possible mechanism for the ion exchange mechanism [19]. According to the proposed scheme, D164 serves as the Na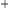 -binding site while D163 serves as the molecular “switch” between the alternating conformations of the protein. Specifically, when D163 is deprotonated it is accessible to the periplasm and D164 is accessible to the cytoplasmic side of the protein. Conversely, when D163 is neutral it is accessible to the cytoplasm, while D164 is accessible to the periplasm. This concerted conformational change accounts for the pumping function of the protein, as well as its experimentally determined electrogenic stoichiometry [20]: two protons pumped into the cell for each Na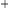 ion transported out.

NhaA was found to be very selective to Na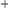, as well as Li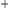 (for a review see [21]), allowing it to detoxify the cell in case of Li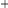 poisoning [22]. Yet, the mechanism of NhaA selectivity is still elusive. How does it permit the passage of only Na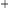 and Li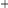 and excludes translocation of other cations? The most straightforward and intuitive explanation is that the selectivity is determined at the first stage of the transport cycle. Specifically, the protein only binds Na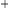 and Li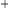 and therefore cannot transport any other ion. Herein, using both computational and experimental approaches, we challenge the above explanation, arriving at a surprising result. The molecular basis for ion selectivity in the Na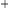/H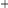 antiporter is not at the ion binding stage. The ion binding site around D164 is promiscuous, capable of binding various alkali ions with no particular preference. However, only Na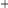 and Li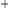 bind the protein in a functional way that allows transport.

## Results

The first stage in the transport process is most likely the binding of the substrate to the protein. Hence, we set out to calculate the free energy profile of this reaction using MD simulations. We then proceeded to experimentally substantiate the computational results. Finally, after having confirmed both experimentally and computationally that binding alone cannot be responsible for the selectivity mechanism of the antiporter, we returned to the simulations in order to gain further insights into potential selectivity source.

### MD simulations

A series of MD simulations of up to 0.1 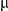
s of the *Escherichia coli* Na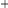/H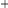 antiporter NhaA in a hydrated lipid bilayer was performed. See Figure S1 for a general overview of the simulation system. In these simulations, the protein was stable, exhibiting low root mean square deviation (RMSD) of its backbone atoms and maintained its secondary structure (see Figure S2). To ensure selection of a representative structure, a cluster analysis was performed to select a conformation for further analyses.

### Potential of mean force

In order to computationally compare the binding of different ions to the antiporter we computed the potential of mean force (PMF) of the process. A PMF is defined as the change in free energy as a function of a particular reaction coordinate. Since the membrane plane coincided with the 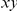 plane in the simulation system, the reaction coordinate was simply the movement of the ion along the 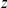 axis from the cytoplasm through the vestibule ending at the binding site (residue D164), in line with the physiological Na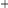 uptake process. To obtain sufficient sampling in thermodynamically unfavorable regions of the protein, we used the umbrella sampling formalism [23]. Specifically, multiple simulations were conducted whereby the only difference between them was the position of the ion along the 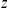 axis. In each of these vertical “slabs” the ion was restrained to the 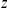 axis by a harmonic restraint but was free to move in the 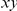 plane. The different slabs were then combined to yield the unbiased PMF using the standard weighted histogram analysis method (WHAM) [24].

**Figure 1:**
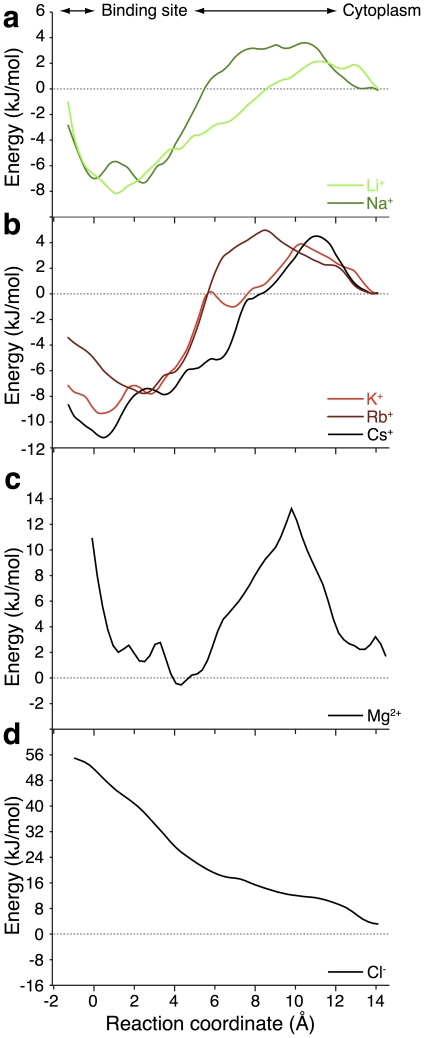
Free energy profiles of cation binding. a. The PMF profiles of Li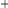 (green) and Na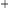 (dark green) ions along the central axis leading into the binding site of NhaA. The reaction coordinate (*z*) starts from the center of the membrane (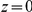) and stretches to the cytoplasm. b. Similar PMF analysis for K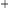 (red), Rb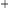 (dark red) and Cs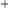 (black). Charts c and d are similar PMF profiles, but for Mg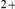 and Cl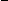, respectively.

The PMF profiles for Na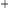 and Li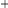 along their uptake pathways are shown in Figure 1 a. For both ions, there are small energy barriers (2–4 kJ/mol) upon movement into the protein that lead to an energy well (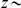
1 Å). Upon inspection of the protein, the energy well corresponds to the location of the putative ion binding site at residue D164. The magnitudes of the energy troughs (7–9 kJ/mol) correspond to the experimentally measured apparent affinity constants for Na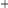 (11–180 mM) and Li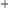 (7–50 mM). The affinity range corresponds to measurements obtained at basic or neutral pH, respectively [25]. Taken together, the PMF analysis for Na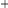 and Li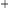 agrees with experimental data, both in terms of the location of the binding site, as well as the magnitude of the binding affinity.

We then proceeded to calculate the PMF curves for K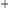, Rb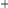 and Cs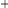, three alkali ions that are not transported by the antiporter. To our surprise, the PMF profiles of K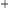, Rb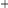 and Cs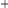 shown in Figure 1 b, are qualitatively similar to that of Li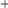 and Na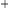 (Figure 1 a), while the quantitative differences are not large enough to account for the fact that the latter ions are substrates of the pump while the former are not.

Finally, we also calculated the PMF profile of a bivalent cation (Mg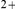) and of an anion (Cl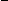), shown in Figures 1 c–d. Both PMF profiles are substantially different from that of the alkali ions and indicate that the two ions are not capable of binding to the antiporter, as expected. Specifically, the PMF analysis of Mg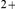 exhibits a high energy barrier upon binding (ca. 14 kJ/mol) without a negative energy trough at the binding site. In contrast, the PMF curve of Cl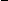 rises continuously upon entry into the protein, most likely due to electrostatic repulsion between the negatively charged anion and the acidic binding pocket at D164.

Taken together, we are left with a surprising result: binding energetics alone cannot be the source of ion selectivity of the antiporter. The PMF analyses for Li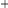 and Na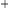 consistently reproduce the location of the binding site of the ions as well as the magnitude of binding affinity. However, the same analysis suggests that other alkali ions are capable of binding the antiporter in a similar fashion. As this result is unexpected, we set forth to confirm it experimentally.

### Experimental analysis of ion binding

**Figure 2:**
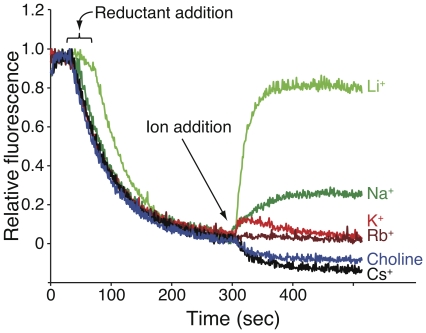
Experimental analysis of antiporter activity using the quinacrine fluorescence quenching method. Everted membrane vesicles activity was determined using acridine orange fluorescence to monitor 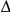
pH. Data of typical measurements are shown. At the onset of the reaction, succinic acid (250 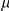
M) was added to energize the vesicles and fluorescence was recorded until a steady state level of 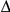
pH (100% quenching) was reached. NhaA activation level was defined as the percentage of dequenching at steady state after adding the respective ion, from those 100%. The concentration of Na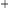 or Li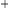 in the experiment was 3.2 mM, while 20 mM was used for the other cations. Higher concentrations of the K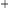, Rb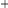 and Cs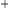 were used to better demonstrate the fact that they are not proper substrates of the transporter.

Measurement of antiporter activity may be conducted in everted membranes using the acridine orange fluorescence quenching method [26], [27]. In brief, when a reductant (*e.g.* succinate) is added to an everted membranes preparation, the respiratory chain acidifies the vesicle interior leading to the accumulation of the mild base acridine orange. The accumulation of the fluorophore leads to self quenching which can be reversed by any protein that can transfer protons out of the vesicles. Since H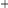 transport is coupled to the counter transport of an alkali ion (*e.g.* Na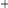), antiporter activity can readily be measured by the ability of the ion to restore fluorescence. For example, analyses of the five different alkali cations using the aforementioned method (Figure 2) clearly demonstrate that only Na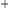 and Li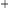 are transported, with Li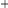 resulting in higher activity, in accordance with [25]. On the other hand, the antiporter shows no activity upon the addition of K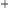, Rb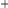 or Cs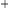, thereby confirming that niter of these ions are substrates of the protein (Choline is used as a negative control).

We could now proceed to experimentally substantiate the computational results which indicate that all five alkali ions bind to the antiporter despite the fact that only Li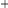 and Na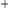 are proper substrate which are subsequently transported. In other words, the computational results posit that K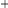, Rb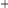 and Cs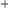 may inhibit the pumping of Na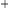 and Li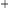 due to competition for the same binding site.

**Figure 3:**
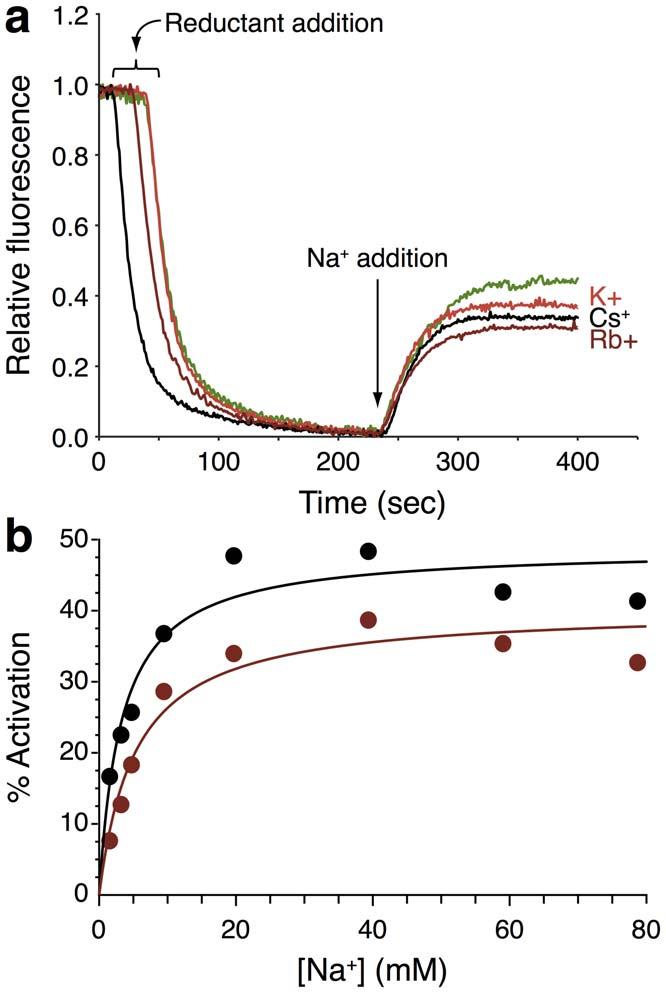
Inhibition of NhaA activity in everted membrane vesicles. See Figure 2 for details. a. Pumping measurements were taken for the native state (green, without inhibition) or at the presence of 20 mM K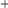 (red), Rb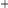 (dark red) or Cs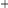 (black). b. Michaelis-Menten kinetic fit. Activation of NhaA was measured at different Na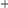 concentrations, in the absence (black) or presence of 20 mM Rb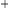 (dark red). The averages (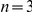) are shown in circles, while the fit is shown in solid lines, error bars were too small to be visible on the graph and hence were omitted.

Indeed, results shown in Figure 3 a are in full agreement with the above hypothesis: the presence of either K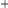, Rb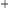 or Cs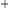 causes an inhibitory effect on the Na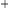 pumping activity of NhaA. Addition of either K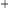, Rb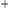, Cs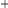 or choline alone does not result in any antiporting (Figure 2). Furthermore, we were able to show that the ions bind in the same location with a detailed Michaelis-Menten kinetic analysis (Figure 3 b). Specifically, we measured the pumping activity of the protein as a function of different Na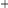 concentrations yielding an apparent 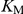 of 3.31
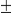
0.54 mM and a 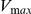 of 48.50
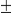
2.0 (a.u.). When the same experiment was conducted in the presence of 20 mM Rb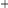, the 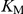 increased by 63% to 5.40
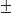
0.83 mM. In contrast, the apparent 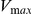 remained practically unchanged with a drop of only 17% to 40.30
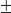
2.3 (a.u.). These trends, of a significant increase in 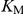 accompanied by a mild change in 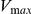 were repeated in several cases, including the use of Li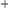 as a substrate (data not shown). These results imply that Rb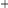 acts as a competitive inhibitor (at least in part) as further discussed below.

Thus, two lines of evidence, *in silico* and *in vitro* experiments, point to the same conclusion: the antiporter binds all alkali ions competitively, yet is capable of completing a pumping cycle only for Li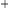 and Na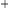. Therefore, one can rule out binding as the source of selectivity of the antiporter. Faced with the above conclusion, we set forth to examine features that might distinguish the “futile” binding ions (K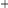, Rb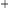 and Cs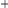) from the productive ones (Li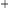 and Na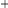). Hence, we decided to examine the binding process in detail and in particular focus on the hydration state of each ion during the binding process.

### Hydration analysis

In order to examine the hydration state of the ions as a function of their penetration into the protein, we made use of the slabs from the PMF analysis. For each of these slabs a water radial distribution function (RDF, 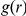) was computed. Subsequently, all slabs were pooled together in order to construct a 3D hydration profile (Figure S3). This analysis describes how the water density varies as a function of the distance from the ion, and as a function of the location of the ion along the 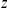 axis.

**Figure 4:**
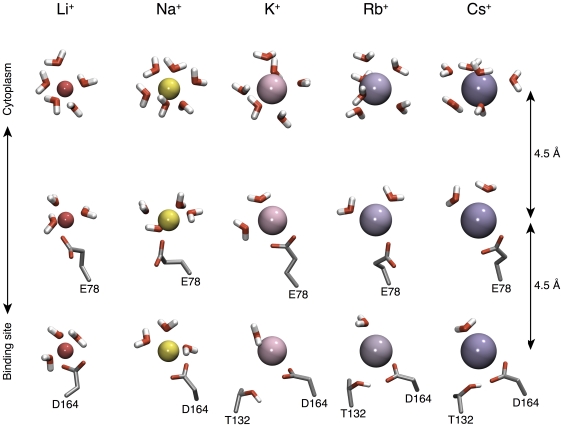
Snapshots of hydration state during the cation binding process. Detailed snapshots of the hydration layers of the various alkali ions as a function of their penetration into the protein. For each of the five ions, three representative snapshots are presented, vertically separated by 4.5 Å.

Our results show that Li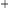 and Na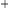 retain their two solvation layers almost fully unimpaired throughout the entire trajectory of the ion until reaching the binding site of the protein. In contrast, all the larger alkali ions, K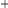, Rb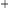 and Cs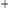 experience a marked reduction in their hydration upon entry into the ion binding site. Snapshots of this process can be seen in Figure 4, whereby one can observe a decrease in the number of water molecules bound to K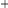, Rb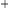 and Cs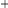 as they approach the ion binding site. In contrast, the hydration of Li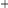 and Na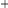 does not change upon binding to the protein, noting that the analysis refers to the section around the ions that is facing the cytoplasm.

## Discussion

The current study poses the following question: What is the selectivity mechanism of *Escherichia coli's* NhaA, the archetypal Na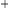/H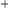 antiporter? The protein is capable of transporting only the small alkali ions, Na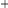 and Li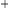, and is incapable of pumping the larger K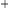, Rb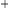 and Cs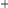. In order to answer the above question, we first attempted to figure out when is selectivity attained. Specifically, the transport cycle might contain numerous sequential steps. Therefore, overall selectivity will arise even if only one of the transport steps is selective for one ion over another. As such, the most likely step in the transport cycle to attain selectivity is the first stage in the process - binding of the substrate.

To examine the selectivity of binding, we performed MD simulations of NhaA embedded in a hydrated lipid bilayer. Then, using umbrella sampling, we obtained well converged PMF curves, ranging from the binding site of the protein, through its cytoplasmic funnel and toward the bulk, for a series of alkali ions. The construction of PMF profiles for Li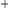, Na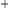, K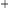, Rb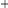 and Cs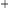 ions provided an opportunity for a comparative overview, and consequently into the selectivity of the binding process.

As stated above, the PMF analyses of Li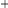 and Na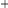 fit to the experimental data regarding the location of the binding site as well as the magnitude of binding affinity. However, the surprising finding is that the PMF binding curves for K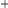, Rb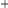 or Cs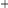 are similar to those obtained for Li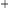 and Na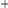. Following the PMF profiles, indicating that all alkali ions tested are capable of binding NhaA, we hypothesized that if indeed binding of K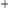, Rb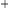 or Cs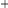 occurs, but antiporting does not, then these ions should serve as competitive inhibitors of Na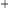 (or Li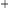) transport.

In line with this hypothesis, we performed experimental analyses in everted vesicles to verify the above conclusions. Indeed we found that the three large alkali ions, K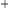, Rb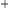 or Cs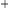 inhibit Na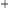 and Li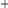 transport (Figure 3). Furthermore, we were able to show that the inhibition is competitive using Michaelis-Menten analysis. Were the system ideal, one would expect a constant 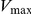 and an increased 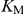 as in the case of a classic competitive inhibition. However, the current assay is indirect and hence reflects internal deviations that stem from the measurement technique. Nonetheless, at high Na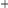 concentrations, it nearly out-competes Rb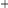, as expected in the case of competitive inhibition. Therefore, we propose that Rb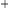 may serve as a competitive inhibitor since we observe a significant increase of the 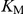 values whereas the decline of the 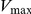 is less than 20%. Hence we suggest, based on *in silico* and *in vitro* experiments, that binding energetics alone, cannot be the source of antiporter selectivity, and that the binding site is not highly specific.

We propose that “binding” is not the relevant term to examine in the case of the Na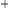/H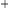 antiporter, but rather “functional binding”. All alkali ions bind the protein at the same site (D164), yet only Na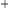 and Li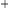 bind in a manner which allows them to be transported. Since these ions are the only ones that retain their solvation in our analysis, we propose that this differentiates them from other alkali ions.

**Figure 5:**
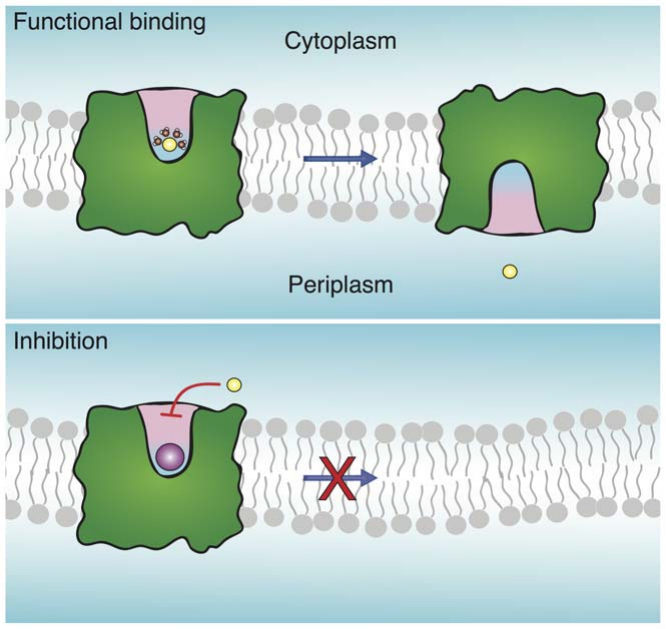
Schematic representation of the selectivity mechanism of NhaA. Top: A productive ion (*e.g.* Na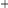, in yellow) is bound to the protein and is capable of being transported. Bottom: A counterproductive ion (*e.g.* Rb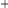, in purple) is bound, cannot be transported and prevents substrate from binding.

Based on the results presented in the current study and previous works [16], [18], [19], we present a model for NhaA selectivity, shown schematically in Figure 5. Li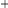 or Na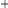 bind to D164 driving subsequent protein conformational changes that eventually lead to their release to the periplasm. However, when K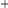, Rb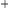 or Cs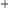 bind to D164, a transport cycle does not ensue, but the binding site is occupied. Therefore, as the results show, binding of K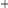, Rb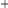 or Cs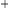 to D164 exerts an inhibitory effect, preventing other productive cations from binding to the protein.

Ion selectivity has been a subject of considerable research [1]–[12]. Pioneering studies by Mullins [28] and Bezanilla & Armstrong [29] suggested that penetration of an ion through a membrane requires that protein groups replace the water molecules forming the ion solvation shell. The ion selectivity in K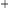 channels was first structurally investigated by MacKinnon and co-workers [30] and then computationally by Roux and co-workers (for review see [8]). Apparently the KcsA channel does not select for K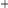 ions by providing a binding site of an appropriate fixed cavity size; rather, selectivity arises directly from the intrinsic local physical properties of the ligands coordinating the cation at the binding site. Additionally, the selectivity for conducting Ca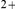 ions in the Ca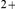 pump is enabled by the ability of the protein to undergo particular conformational changes [31], [32]. In the presence of Ca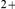, the selectivity filter sites in the Ca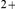 pump fit around Ca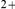 ions and not other cations whereas the filter adopts a conductive conformation and specific coordination.

Our results are compatible with all these studies and may complement them. We agree with the suggestions of Mullins [28] and Bezanilla & Armstrong [29] and add that a functional binding of an ion to NhaA involves a retainment of its solvation shells. Our study is in accord with the mechanism of selectivity at the KcsA channel [8] and the Ca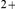 pump [31], [32] as well since we claim that the selectivity of NhaA is not determined simply by size.

Finally, it is tempting to speculate that other antiporters might share the same selectivity mechanism. For example, two distinct orthologs of Na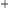/H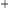 seem to bind Rb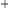 : Nha1p, the budding yeast's Na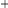/H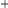 antiporter, was found to have a low affinity to Rb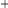 [33]. Similarly, NHE, an ortholog of NhaA from rat pancreas membranes, is inhibited by Rb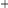 [34]. In conclusion, two lines of analyses, *in silico* and *in vitro* converge at a consistent picture: The binding site of NhaA is permissive and allows binding of all alkali ions tested, and binding alone is insufficient to account for selectivity.

## Materials and Methods

### Simulation system set up

The x-ray structure of the *Escherichia coli's* NhaA, determined at 3.45 Å, was downloaded from the PDB (entry 1ZCD [16]). A pre-equilibrated 1-palmitoyl-2-oleoyl-sn-glycero-3-phosphoethanolamine (POPE) bilayer [35], which initially contained 340 lipids and 6729 molecules of SPC water [36], trimmed to 244 lipids and 4237 water molecules, and further equilibrated for 2 ns, was used for the membrane in which the protein was embedded. The antiporter's rough axis was aligned perpendicular to the membrane plane and all colliding lipid and water molecules, within 2 Å of the protein, were manually removed (down to 153 lipids and 4032 water molecules). The system's total charge was neutralized by adding K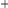 and Cl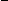 ions to a final concentration of 0.1 M, replacing randomly distributed water molecules. The system was subjected to rigorous energy minimization using the steepest descent algorithm and a tolerance of 1000 kJ

mol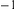 
nm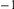, followed by a minimization using the conjugated gradient algorithm with a sequential decreasing convergence from 100 to 10 kJ

mol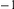 
nm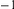. Then, an equilibration stage under positional restraints using a harmonic force constant was conducted. The equilibration procedure began with a force constant of k = 1000 kJ

mol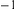 
nm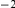 for 100 ps, then a force constant of k = 500 kJ

mol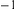 
nm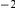 for 100 ps, and another 100 ps of an unrestrained MD run. This allowed the lipids and water to pack more tightly around the protein, and enabled the protein gradual relaxation in the membrane. After the positional restraint equilibration, the system was submitted for unbiased MD runs of up to 0.1 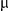
s.

### MD details

The simulations were conducted using the GROMACS package [37], [38], employing an extended version of the GROMOS53a6 force field [39]. All simulations were conducted using the LINCS algorithm [40] to constrain bond lengths and angles of hydrogen atoms, allowing a time step of 2 fs. Simulations were run using Berendsen temperature coupling at 310 K employing a coupling constant of 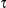 = 0.1 ps. Pressure was kept constant at 1 bar by applying semi-isotropic coupling with a coupling constant of 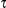 = 1 ps, differentiating the 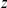 axis (the membrane normal). A cutoff of 1.2 nm was used for van der Waals interactions, and long range electrostatic interactions were computed using the PME method [41].

### Potential of mean force (PMF)

PMF calculations were done using the umbrella sampling formalism [42]. After reaching equilibrium and observing that water molecules entered the protein's vestibules, a selected ion (Li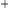, Na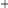, K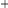, Rb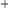 or Cs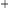) was inserted within the putative binding site of the protein by manually placing the ion to a non-clashing proximity. The Lennard-Jones (LJ) parameters for Li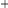 were taken from [43] and those of Rb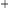 and Cs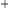 were adopted from OPLS and converted to the GROMOS53a6 force field.

The coordinate for the umbrella sampling windows, taken at 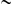
1 Å intervals, was the 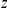 axis from the cytoplasmic vestibule of the antiporter, to the cytoplasmic bulk, setting 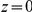 at the center of the membrane. For each window the system was minimized to allow the ion to move laterally in the *xy* plane prior to the 1 ns production run. Unbiasing and integration were done using the Weighted Histogram Analysis Method (WHAM) [24]. The energy curves were vertically fitted so that they superimpose where the ion is outside of the vestibule (set to zero energy on the profiles).

### Radial Distribution Function

Radial Distribution Function, RDF, *g*(r), was calculated for the investigated cations for each of the windows that were constructed for the PMF analysis. This yielded 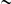
20 RDF curves for each ion in relation to the water around it. All the curves for each ion were plotted together to construct a 3D graph that describes how the water density varies as a function of the distance from the tested ion and of the location of the tested ion along the 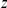 axis as well.

### Visualization and analysis

The simulations were visualized with the Visual Molecular Dynamics (VMD) program [44]. The analyses were conducted using in-house VMD Tcl scripts, in-house purpose written perl scripts, and the GROMACS analysis package tools.

### Bacterial strains and plasmids

The *Escherichia coli* strain used for growth and expression was KNabc (TG1 derivative, 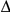
nhaA 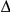
nhaB 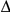
chaA [45]) which is strongly inhibited by NaCl and LiCl. All sub-cloning were done using a pBR322-derived plasmid regulated under NhaR [46] (a kind gift from Prof. E. Padan, The Hebrew University of Jerusalem, Israel) containing the NhaA gene or no gene for control. Plasmid amplification was done in DH5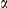 cells. Growth media was Lysogeny Broth (LB) [47], unless otherwise stated. Antibiotics concentration was 100 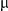
g/ml ampicillin.

### Vesicles and fluorescence quenching

Everted membranes of *Escherichia coli* were produced using the technique introduced by Rosen and Tsuchiya [48] with the following steps: lysis buffer used contained 21% sucrose, 15 mM Tris/HCl buffer at pH 7.5 and 150 mM choline-chloride. Bacteria were grown overnight in LB medium, washed three times in lysis buffer, suspended in 5 ml/gr and broken once in a French press at 900 psi (valve pressure). Broken bacteria solution was centrifuged at 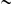
3000 g for 20 minutes following by centrifugation of the supernatant at 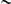
340,000 g for 20 minutes. The final pellet, containing the vesicles, was resuspended in lysis buffer with 1 ml/gr of original dry bacteria, and frozen in liquid nitrogen.

NhaA activity was measured by the quinacrine fluorescence quenching method [26], [27], using lysis buffer and 2–5 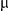
M of Acridine Orange (N,N,N′,N′-Tetramethylacridine-3,6-diamine). Succinic acid (250 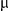
M) or D-lactate (0.8 mM) were used to energize the vesicles. 100% quenching was defined as the difference in fluorescence between prior to addition of a reductant and after a steady state was achieved. NhaA activation level was defined as the fraction of dequenching at steady state after adding Na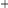 or Li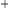, from those 100%. Where potential inhibitors were added (K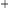, Rb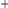 or Cs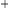), addition of 20 mM (unless otherwise stated) inhibitor was made before starting the fluorescence reading. Otherwise, the same concentration of choline-chloride was added. Fluorescence was excited at 366 nm and emission was read at 531 nm using a FluoroMax-3 spectrofluorometer (HORIBA Jobin Yvon).

Kinetic analysis was done using fluorescence quenching results as presented above. Activations under different ion concentrations were plotted together using a Michaelis-Menten simple enzyme kinetic model [49]. Regression was performed using the nonlinear least sum of squares technique. Statistical data were obtained using the bootstrapping method on the entire dataset, consisting of at least 3 repeats for each data point. The bootstrapping process was done using over 1000 cycles and its convergence was evaluated using standard statistical procedures.

## Supporting Information

Figure S1
**The simulation system presenting the structure of the Na** 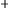 **/H** 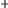 **antiporter embedded in a lipid bilayer.** The protein is shown in a cartoon representation and the 12 TMSs are labeled in Roman numerals. Some lipids were omitted for visual clarity.(TIFF)

Figure S2 **Protein stability during the simulations.** a. Backbone RMSD analysis relative to the x-ray structure as a function of the simulation time. b. Secondary structure content of the protein relative to the initial structure during the course of the simulations. The total secondary structure is in black while the helical content is in gray.
(TIFF)

Figure S3
**Hydration profiles during cation binding.** Water radial distribution, *g*(r), of different ions (Li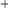, Na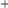, K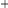, Rb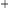 and Cs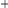) as a function of the distance from the tested ion and of the location of the tested ion along the 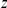 axis (the membrane normal) as well. The first solvation shell of each ion is located at the right side of each panel and is followed by the second solvation shell that is drawn to its left. The front slice is closest to the ion binding site, while the depth represents the ion being further out into the cytoplasmic bulk. The color ranges from blue to red and represents low to high 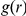, respectively.(TIFF)

## References

[1] Corry B (2006). Understanding ion channel selectivity and gating and their role in cellular signalling.. Mol Biosyst.

[2] Gouaux E, Mackinnon R (2005). Principles of selective ion transport in channels and pumps.. Science.

[3] Hachez C, Chaumont F (2010). Aquaporins: a family of highly regulated multifunctional channels.. Adv Exp Med Biol.

[4] Jensen ML, Schousboe A, Ahring PK (2005). Charge selectivity of the cys-loop family of ligandgated ion channels.. J Neurochem.

[5] Keramidas A, Moorhouse AJ, Schofield PR, Barry PH (2004). Ligand-gated ion channels: mechanisms underlying ion selectivity.. Prog Biophys Mol Biol.

[6] Miloshevsky GV, Jordan PC (2004). Permeation in ion channels: the interplay of structure and theory.. Trends Neurosci.

[7] Moomaw AS, Maguire ME (2008). The unique nature of mg2+ channels.. Physiology (Bethesda).

[8] Noskov SY, Roux B (2006). Ion selectivity in potassium channels.. Biophys Chem.

[9] Owsianik G, Talavera K, Voets T, Nilius B (2006). Permeation and selectivity of trp channels.. Annu Rev Physiol.

[10] Roux B (2005). Ion conduction and selectivity in k+ channels.. Annual Review of Biophysics and Biomolecular Structure.

[11] Yamaoka K, Vogel SM, Seyama I (2006). Na+ channel pharmacology and molecular mechanisms of gating.. Curr Pharm Des.

[12] Zhou HX, McCammon JA (2010). The gates of ion channels and enzymes.. Trends Biochem Sci.

[13] West IC, Mitchell P (1974). Proton/sodium ion antiport in Escherichia coli.. Biochem J.

[14] Orlowski J, Grinstein S (2004). Diversity of the mammalian sodium/proton exchanger SLC9 gene family.. Pugers Arch.

[15] Karpel R, Olami Y, Taglicht D, Schuldiner S, Padan E (1988). Sequencing of the gene ant which affects the Na^+^/H^+^ antiporter activity in Escherichia coli.. J Biol Chem.

[16] Hunte C, Screpanti E, Venturi M, Rimon A, Padan E (2005). Structure of a Na^+^/H^+^ antiporter and insights into mechanism of action and regulation by pH.. Nature.

[17] Padan E (2008). The enlightening encounter between structure and function in the NhaA Na^+^-H^+^ antiporter.. Trends Biochem Sci.

[18] Inoue H, Noumi T, Tsuchiya T, Kanazawa H (1995). Essential aspartic acid residues, Asp-133, Asp-163 and Asp-164, in the transmembrane helices of a Na^+^/H^+^ antiporter (NhaA) from Escherichia coli.. FEBS Lett.

[19] Arkin IT, Xu H, Jensen MØ, Arbely E, Bennett ER (2007). Mechanism of Na^+^/H^+^ antiporting.. Science.

[20] Taglicht D, Padan E, Schuldiner S (1993). Proton-sodium stoichiometry of NhaA, an electrogenic antiporter from Escherichia coli.. J Biol Chem.

[21] Padan E, Tzubery T, Herz K, Kozachkov L, Rimon A (2004). NhaA of Escherichia coli, as a model of a pH-regulated Na^+^/H^+^ antiporter.. Biochim Biophys Acta.

[22] Inaba K, Kuroda T, Shimamoto T, Kayahara T, Tsuda M (1994). Lithium toxicity and Na^+^(Li^+^)-H^+^ antiporter in Escherichia coli.. Biol Pharm Bull.

[23] Dickson A, Dinner AR (2010). Enhanced sampling of nonequilibrium steady states.. Annu Rev Phys Chem.

[24] Kumar S, Bouzida D, Swendsen R, Kollman P, Rosenberg J (1992). The Weighted histogram analysis method for free-energy calculations on biomolecules .1. The method.. J Comput Chem.

[25] Zuber D, Krause R, Venturi M, Padan E, Bamberg E (2005). Kinetics of charge translocation in the passive downhill uptake mode of the Na+/H+ antiporter NhaA of Escherichia coli.. Biochim Biophys Acta.

[26] Schuldiner S, Rottenberg H, Avron M (1972). Determination of pH in chloroplasts. 2. Fluorescent amines as a probe for the determination of pH in chloroplasts.. Eur J Biochem.

[27] Schuldiner S, Fishkes H (1978). Sodium-proton antiport in isolated membrane vesicles of Escherichia coli.. Biochemistry.

[28] Mullins LJ (1959). The penetration of some cations into muscle.. J Gen Physiol.

[29] Bezanilla F, Armstrong CM (1972). Negative conductance caused by entry of sodium and cesium ions into the potassium channels of squid axons.. J Gen Physiol.

[30] Doyle DA, Morais Cabral J, Pfuetzner RA, Kuo A, Gulbis JM (1998). The structure of the potassium channel: molecular basis of k+ conduction and selectivity.. Science.

[31] Toyoshima C, Nakasako M, Nomura H, Ogawa H (2000). Crystal structure of the calcium pump ofsarcoplasmic reticulum at 2.6 Å resolution.. Nature.

[32] Toyoshima C, Nomura H (2002). Structural changes in the calcium pump accompanying the dissociation of calcium.. Nature.

[33] Ohgaki R, Nakamura N, Mitsui K, Kanazawa H (2005). Characterization of the ion transport activity of the budding yeast Na^+^/H^+^ antiporter, Nha1p, using isolated secretory vesicles.. Biochim Biophys Acta.

[34] Anderie I, Thévenod F (1996). Evidence for involvement of a zymogen granule Na^+^/H^+^ exchanger in enzyme secretion from rat pancreatic acinar cells.. J Membr Biol.

[35] Tieleman DP, Berendsen HJ (1998). A molecular dynamics study of the pores formed by Escherichia coli OmpF porin in a fully hydrated palmitoyloleoylphosphatidylcholine bilayer.. Biophys J.

[36] Berendsen H, Postma J, van Gunsteren W, Hermans J, Pullman B (1981). Intermolecular forces: Interaction models for water in relation to protein hydrationInteraction models for water in relation to protein hydration..

[37] Van Der Spoel D, Lindahl E, Hess B, Groenhof G, Mark AE (2005). GROMACS: fast, exible, and free.. J Comput Chem.

[38] Berendsen H, van der Spoel D, vandrunen R (1995). GROMACS - a message - passing parallel molecular-dynamics implementation.. Comp Phys Comm.

[39] Siu SWI, Vácha R, Jungwirth P, Böckmann RA (2008). Biomolecular simulations of membranes: physical properties from different force fields.. J Chem Phys.

[40] Hess B, Bekker H, Berendsen H, Fraaije J (1997). LINCS: A linear constraint solver for molecular simulations.. J Comput Chem.

[41] Darden T, York D, Pedersen L (1993). Particle mesh Ewald: an N-log(N) method for Ewald sums in large systems.. J Chem Phys.

[42] Torrie GM, Valleau JP (1977). Nonphysical sampling distributions in Monte carlo free-energy estimation: umbrella sampling.. J Comput Phys.

[43] Impey R, Sprik M, Klein M (1987). Ionic solvation in nonaqueous solvents - The structure of Li^+^ and Cl^−^ in methanol, ammonia, and methylamine.. J Am Chem Soc.

[44] Humphrey W, Dalke A, Schulten K (1996). VMD: visual molecular dynamics.. J Mol Graph.

[45] Nozaki K, Inaba K, Kuroda T, Tsuda M, Tsuchiya T (1996). Cloning and sequencing of the gene for Na^+^/H^+^ antiporter of Vibrio parahaemolyticus.. Biochem Biophys Res Commun.

[46] Rahav-Manor O, Carmel O, Karpel R, Taglicht D, Glaser G (1992). NhaR, a protein homologous to a family of bacterial regulatory proteins (LysR), regulates nhaA, the sodium proton antiporter gene in Escherichia coli.. J Biol Chem.

[47] Bertani G (1951). Studies on lysogenesis. I. The mode of phage liberation by lysogenic Escherichia coli.. J Bacteriol.

[48] Tsuchiya T, Rosen BP (1975). Characterization of an active transport system for calcium in inverted membrane vesicles of Escherichia coli.. J Biol Chem.

[49] Michaelis L, Menten ML (1913). Kinetics of Invertase Action.. Biochem Z.

