# Supplementary figures and images for: Promiscuous Binding in a Selective Protein: The Bacterial Na+/H+ Antiporter
Source: PLoS One. 2011 Oct 12;6(10):e25182. doi: 10.1371/journal.pone.0025182 (PMC3192041; doi:10.1371/journal.pone.0025182)

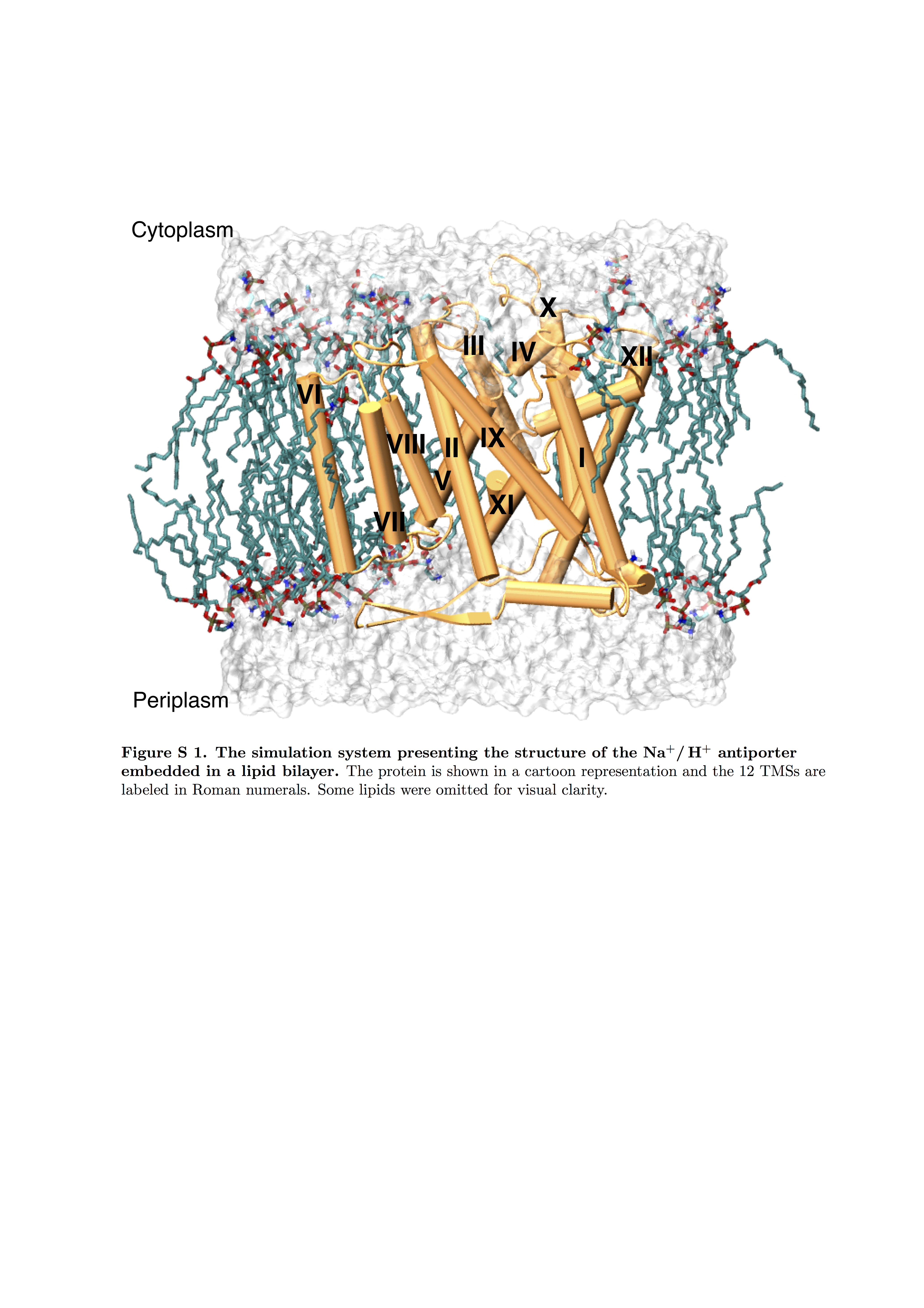

Supplement: Figure S1 — The simulation system presenting the structure of the Na /H antiporter embedded in a lipid bilayer. The protein is shown in a cartoon representation and the 12 TMSs are labeled in Roman numerals. Some lipids were omitted for visual clarity. (TIFF) [file pone.0025182.s001.tiff]

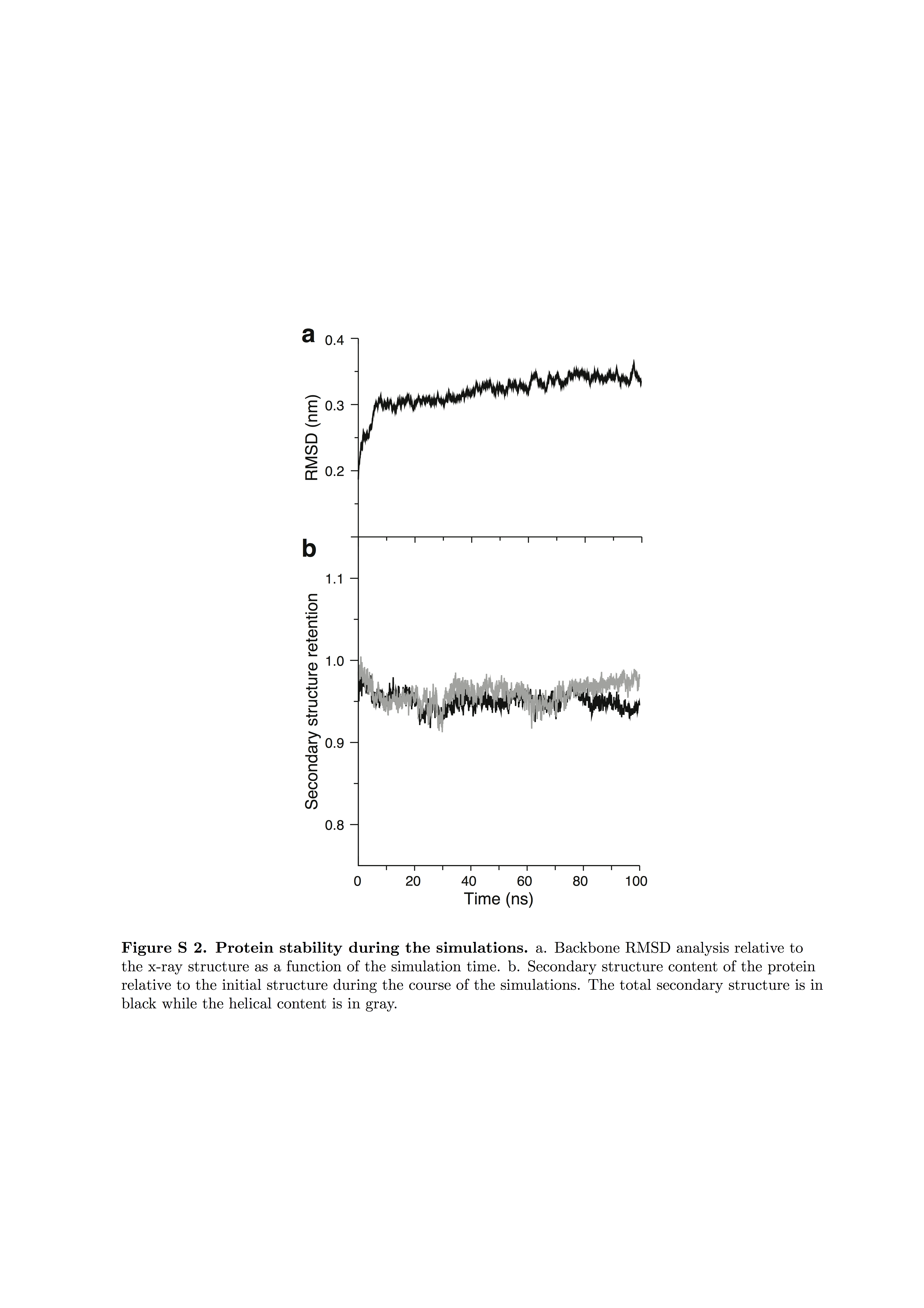

Supplement: Figure S2 — Protein stability during the simulations. a. Backbone RMSD analysis relative to the x-ray structure as a function of the simulation time. b. Secondary structure content of the protein relative to the initial structure during the course of the simulations. The total secondary structure is in black while the helical content is in gray. (TIFF) [file pone.0025182.s002.tiff]

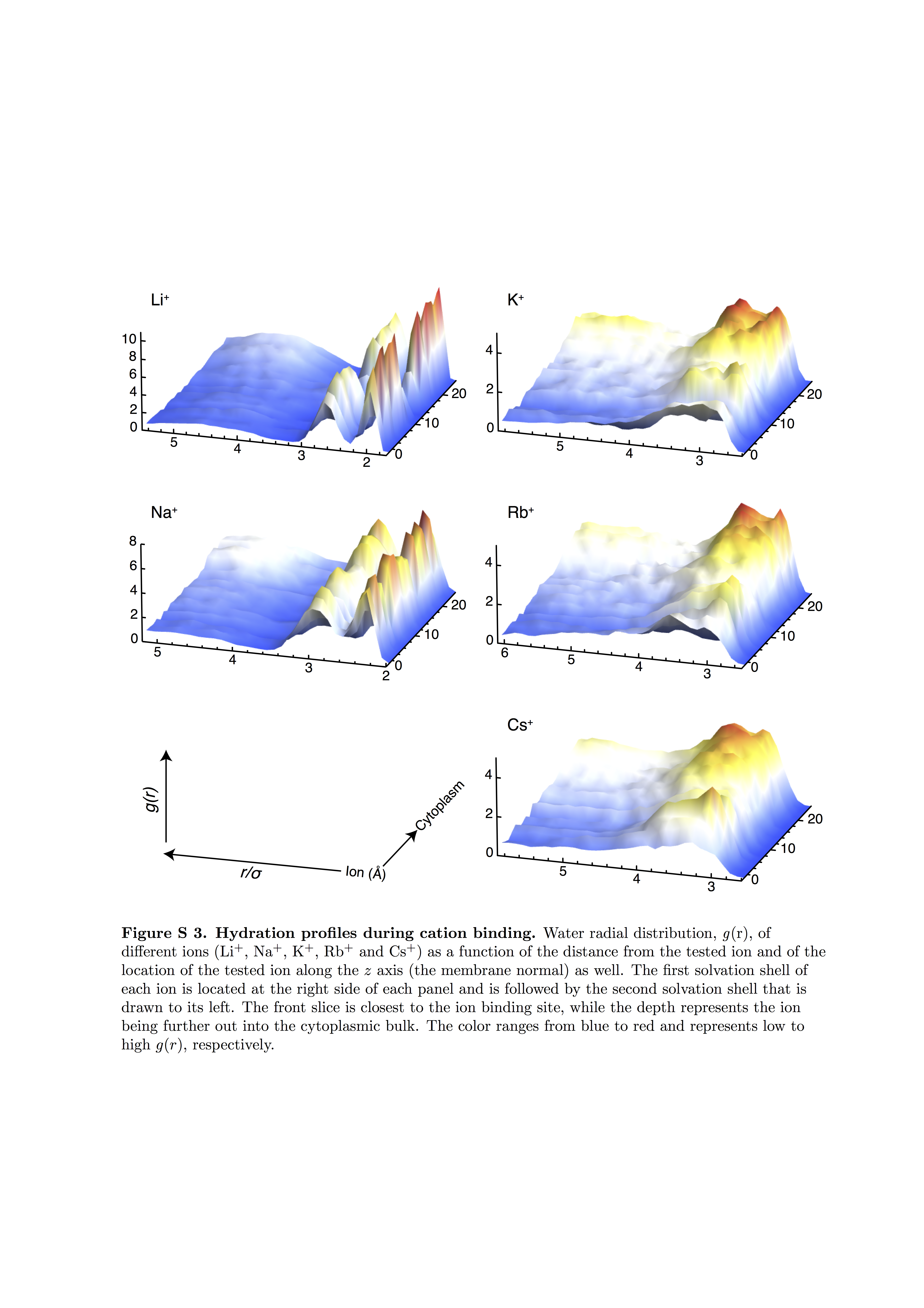

Supplement: Figure S3 — Hydration profiles during cation binding. Water radial distribution, g(r), of different ions (Li, Na, K, Rb and Cs) as a function of the distance from the tested ion and of the location of the tested ion along the axis (the membrane normal) as well. The first solvation shell of each ion is located at the right side of each panel and is followed by the second solvation shell that is drawn to its left. The front slice is closest to the ion binding site, while the depth represents the ion being further out into the cytoplasmic bulk. The color ranges from blue to red and represents low to high , respectively. (TIFF) [file pone.0025182.s003.tiff]
